# The Role of Early Life Environmental Risk Factors in Parkinson Disease: What Is the Evidence?

**DOI:** 10.1289/ehp.7573

**Published:** 2005-05-26

**Authors:** Giancarlo Logroscino

**Affiliations:** Department of Epidemiology, Harvard School of Public Health, Boston, Massachusetts, USA

**Keywords:** early life, Parkinson disease, pesticides, place of birth, smoking, toxicants

## Abstract

Parkinson disease (PD) is of unknown but presumably multifactorial etiology. Neuropathologic studies and animal models show that exposure to environmental neurotoxicants can determine progressive damage in the substantia nigra many years before the onset of clinical parkinsonism. Therefore, PD, like other neurologic diseases related to aging, may be determined by exposures present in the environment early during the life span or even during pregnancy. Recent epidemiologic studies have focused on the possible role of environmental risk factors present during adult life or aging. Smoking and coffee drinking have consistently been identified to have protective associations, whereas roles of other risk factors such as pesticide and infections have been reported in some studies but not replicated in others. Both genetic inheritance and sharing of common environment in the same family explain the increased risk of PD of relatives of PD cases compared with relatives of controls in familial aggregation studies. Much evidence indicates that risk factors that have a long latency or a slow effect could be important for late-onset PD. Further epidemiologic studies are warranted in this area.

Parkinson disease (PD) is of unknown but presumably multifactorial etiology. The main impetus for the environmental causation theory of PD came from the isolation of the chemical compound MPTP (1-methyl-4-phenyl-1,2,3,6-tetrahydropyridine) that was associated with marked parkinsonism in four young adults after using a Demerol derivative intravenously (Langston et al. 1984).

Toxic substances can cause static or progressive damage. The distinction between these two pathogenetic mechanisms is relevant to understanding whether early environmental risk factors play a role in the pathogenesis of PD. MPTP produces both acute and progressive cellular loss in the substantia nigra. MPTP exposure at one point in time can be responsible for a progressive clinical syndrome with an intervening latent period.

The neuropathologic study of the brains of three MPTP-exposed subjects showed microglial activation with an ongoing inflammatory reaction 3–6 years after the toxic exposure (Langston et al. 1999). In each case neuropathologic examination of the brain revealed depletion of pigmented neurons in the substantia nigra but without Lewy bodies, the pathologic hallmark of PD. These findings indicate an active, ongoing neuronal loss, suggesting that an event happening at a single point in time may determine a slowly progressive neurodegenerative process in the substantia nigra (Langston et al. 1999). In monkeys exposed to MPTP 5–14 years before death and free from MPTP exposure for 3 years before death, biopsy data showed reactive microglia and extracellular melanin (McGeer et al. 2003). The results of these two studies demonstrate that toxic exposure many years before the onset of clinical parkinsonism can result in a progressive neuronal loss and a permanent inflammatory reaction in the brain of patients with PD. Consistent with this model, a brain injury could produce a progressive loss of dopaminergic (DA) cells prenatally or early in life. Subsequently, a second toxic insult or normal aging could determine a selective neuro-degenerative process in the substantia nigra (Langston 1990).

Even if sporadic PD is a neurodegenerative disorder that characteristically begins after 50 years of age, the possibility that it could be determined by the early life environment has been proposed in the past (Martyn and Osmond 1995). In this review I focus on published evidence from both experimental and epidemiologic studies of a possible role of early life environmental risk factors for PD.

## Infections in Early Life

Inflammation has been proposed as one of the possible pathogenetic mechanisms for PD (McGeer et al. 2001). The proinflammatory cytokine tumor necrosis factor-α (TNF-α) kills DA neurons *in vitro* and is elevated in the brains of patients with PD (Mogi et al. 1994). Intrauterine infection might lead to nigral cell loss in the fetal brain and a subsequent decrease in DA neurons in the substantia nigra secondary to inflammation. Bacterial vaginosis during pregnancy is characterized by the presence of lipopolysaccharide (LPS), a bacteriotoxin that is an inducer of TNF-α (Thorsen et al. 1998). These compounds are increased in the chorioamniotic environment of infected women and could interfere with the normal development of DA neurons. Ling et al. (2002) injected gravid female rats intraperitoneally with LPS, and the newborn rats were killed 3 weeks after birth. The pre-natal exposure to LPS produced lesions that were specific to DA cells. DA cells were counted in the mesencephalon using tyrosine hydroxylase–immunoreactive (THir) cells as a DA neuron marker, and a significant reduction of cell number was found (27%). The LPS treatment reduced the THir cells, but not microtubule-associated protein-2 (MAP-2), a marker of all neurons. The specificity of the lesions and the timing of the inflammatory response to LPS injection in the fetal brain suggest that LPS might interfere with normal DA neuron development.

These results outline the possibility that, after intrauterine infection, some individuals may be born with limited striatal neurochemical reserves and a reduced DA cell count. This reduced reserve might predispose adults to PD. Perinatal infections may therefore represent risk factors for PD. Most epidemiologic studies on infection during early life and risk for PD were conducted in England. A population-based study was conducted in 42 general practices in eastern Hertfordshire (Martyn and Osmond 1995). PD cases were subjects born after 1910 and diagnosed by a neurologist or a geriatrician, and controls were subjects in the same general practices matched by age and sex (172 cases, 343 controls). Records in the first part of the 20th century were used to obtain information about birth weight and growth during the first year of life. The subjects answered a questionnaire about sibship, position in the birth order, type of housing, other features of the environment in early life, and experience of the common infections of childhood. Subjects were more likely to have suffered croup [odds ratio (OR) = 4.1; 95% confidence interval (CI), 1.1–16.1] or diphtheria (OR = 2.3; 95% CI, 1.1–3.6). The results of this study do not suggest that poor growth in fetal life or infancy is important in the etiology of PD but do suggest that early infection might have a role in susceptibility to PD. A study conducted in a Harvard alumni cohort showed a negative association between measles infection and PD (Sasco and Paffenbarger 1985), and a reduced risk of PD was associated with most childhood viral infections. The authors speculate that a negative history of measles could be a marker for negative influenza history before 1918 and thus a higher risk of infection during the 1918 influenza epidemic because of the lack of partial influenza immunity. The data on infections in these studies were based on recall and therefore should be interpreted with caution (although a negative association in the Harvard study is not what would be expected in presence of recall bias).

Some investigators have examined the relationship between influenza pandemics and PD, testing the hypothesis that PD could follow intrauterine influenza (Mattock et al. 1988). Three groups of PD patients were identified through specialty clinics or general practitioners. Matched controls were selected so that they were born in the same 5-year period as the index patient. For each group and each calendar year, the ratio of parkinsonian to control births was calculated. This risk ratio was used to estimate the relative risk of each cohort developing PD in later life. Calendar years with a PD:control ratio of 1.66 or greater were defined as “cluster years.” Subjects born in 1892, 1904, 1909, 1918, 1919, and 1929 (years encompassing the influenza pandemics of 1890–1930) appeared to have had an increased risk of developing idiopathic PD compared with subjects born in other periods, and four of these years coincide with years of influenza pandemic. The PD:control birth ratio showed a significant correlation with the crude influenza mortality for the year of birth during the period from 1900 to 1930 (Mattock et al. 1988). People born around 1900 had a risk of PD 5 times that of people born around 1930 (Martyn 1997). A study conducted in Aberdeen, Scotland, failed to replicate these results (Ebmeier et al. 1989). A cohort of 243 subjects with PD resident in Aberdeen confirmed a peak of PD births in the year 1902, but there was no association between PD births and influenza deaths. The limited power of this cohort could be an explanation for the negative results. The strong variation of PD risk from year to year of birth shown in some of the previous studies would be consistent with the action during development of an infectious agent, more likely a virus that typically occurs in epidemics.

Season or month of birth could be used as surrogate of viral insult and has been associated with disorders such as schizophrenia (Mortensen et al. 1999). Mattock et al. (1988) reviewed the season of birth of 517 individuals with PD. They showed an excess of births in the period between March and June, with the greatest excess in May. These results were confirmed in a study in Japan on two different cohorts with an excess of PD births in winter and spring (Miura et al. 1987; Torrey et al. 2000).

All these studies were based on the hypothesis that intrauterine influenza infection or other viral insults may be cytotoxic to the developing fetal substantia nigra and could enhance the risk of PD after a latent period (Poskanzer and Schwab 1963; Takahashi and Yamada 2001).

Data on the season of birth are some of the least compelling arguments for a relation between influenza and PD because there are other potential variables that are characterized by a seasonal variation that could be responsible for the observed association. Epidemiologic studies on the relationship between infections early in life and PD are definitively inconclusive.

## Place of Birth

Geographic epidemiology is defined as a description of the spatial pattern of disease occurrence (Elliot et al. 1992). Place of birth can be a surrogate for early-life exposure. State of birth in the United States was studied as an exposure of interest for PD (Betemps and Buncher 1993). Outcome was measured by death, using death certificates from U.S. death information in 1981. The measure of effect was the proportionate mortality ratio (PMR), using all the other deaths in the United States as the comparison group. Potential confounders such as age, gender, and whether the state of birth was the same as the state of death were included in a logistic model. Cerebrovascular disease deaths were used as a negative control group because there is no association that is postulated with childhood exposure; multiple sclerosis was used as a positive control because it has been found to be related to geography in childhood. The results showed a very high PMR for Utah (44.95/10,000), Idaho (37/10,000), Colorado (31/10,000), New Mexico (32/10,000), Kansas (32/10,000), and Nebraska (33/10,000), with a west to east gradient. The lowest PMR was registered in Delaware (7.3/10,0000). However, it is important to mention that PMR can be affected by the distribution of other causes of death. In contrast, a study conducted by Kurtzke and Goldberg (1988) using death certificates showed that age-adjusted death rates of PD presented a north–south gradient, similar to that found for multiple sclerosis. In this study only the state where the PD subject was resident at the time of death was considered in the analysis. Geographic studies based on death certificates may be affected by the underreporting of PD that can be inhomogeneous across different geographic areas. Studies of geographic variation need to be interpreted with caution because many factors beyond environmental exposure could determine the geographic pattern of a disease. Genetic factors and ethnicity are real sources of variation across different regions beyond environmental exposure. Chance, migration, differences in procedures used to diagnose/classify cases, and access to medical care are other possible alternative explanations (Elliot et al. 1992).

## Toxic Exposure Early in Life

The distinction between secondary parkinsonism and PD is critical, particularly when toxicants are the exposure of interest. Therefore, in this context, it is important to underscore that the diagnosis of PD is clinical because no diagnostic biomarker is available. In every study on PD there is some degree of misclassification of cases. In a recent population-based study the initial diagnosis of PD was later confirmed in 83% of 202 diagnosis but rejected in 15% of the initial diagnosis (2% had diagnosis of possible PD; Schrag et al. 2002). The false-positive diagnosis is generally due to vascular parkinsonism, essential tremor, drug-induced parkinsonism, or other neurodegenerative disorders that include parkinsonism as one of the clinical features, such as multisystem atrophy, progressive sopranuclear palsy, and dementia. The use of more stringent criteria causes the rate of false positives to drop down to about 10% (Jellinger 2003). Missing the diagnosis of PD is particularly frequent. Recent door-to-door epidemiologic studies show that almost one of four prevalent cases of PD is detected during the survey in the community (de Rijk et al. 1997). The misclassification of cases if random and not related to the exposure of interest makes the detection of true association more difficult in analytic studies.

Clinical parkinsonism can be caused by a series of exogenous factors such as viruses, toxic substances such as MPTP and manganese, and trauma. These clinical observations suggest that environmental risk factors are important for the etiology of PD. Several environmental toxicants related to occupation in agriculture have been implicated as risk factors in PD. The chemical structure of 1-methyl-4-phenyl-pyridinium (MPP^+^), the toxic metabolite of MPTP, is similar to the herbicide paraquat (PQ; Snyder and D’Amato 1985).

Animal experiments have clearly shown the selective action of PQ on the DA system. Systemic PQ given to mice through repeated intraperitoneal injections induces selective damage of DA neurons in the pars compacta of the substantia nigra (McCormack et al. 2002). The cell loss in this model was dependent on dose and age. The presence of a different effect of the exposure at different ages in this model outlines a possible interaction of toxic environmental exposures with normal aging (McCormack et al. 2002). Chronic, continuous, and systemic exposure to rotenone, commonly used as an insecticide in gardening, selectively inhibits complex I, causing selective degeneration of the nigrostriatal system with accumulation of cytoplasmic inclusions containing ubiquitin and α-synuclein (Betarbet et al. 2000). In mice, PQ exposure determines an up-regulation of α synuclein, accelerating the formation of aggregates in the substantia nigra (Manning-Bog et al. 2002). This confirms previous observations *in vitro* that postulated a selective binding of PQ with partially folded α-synuclein (Uversky et al. 2001).

Toxic exposures that occur early in development could determine long-term pathology in the central nervous system. A model exploring this hypothesis should explore the possibility that early damage to the DA system could result in cell loss or high vulnerability to a second environmental risk factor associated with PD (two-hit model). A similar model was hypothesized for exposure to PQ and the fungicide maneb (MB) during development. The combined action could permanently change the nigrostriatal DA system and enhance its vulnerability to subsequent neurotoxicant challenges. Mice exposed to two neurotoxicants, PQ and MB, and subsequently exposed again as adults presented with the most severe damage. They showed a 70% reduction in motor activity 2 weeks after the last toxic dose (Thiruchelvam et al. 2002). Developmental exposure to PQ or MB alone produced minimal changes, whereas a significant decrease in nigral cell counts was observed after adult exposure. Striatal DA levels were reduced by 37% after only the developmental exposure to PQ plus MB, but after a second exposure during adulthood, striatal DA levels were reduced by 62%. This experiment suggests that the exposure during development to the two toxicants produced a state of masked toxicity that was revealed after adult re-exposure. In another experiment conducted by the same group, pregnant mice were treated with MB or PQ (Barlow et al. 2004). Offspring were normal when evaluated on locomotor activity. Subsequently offspring were treated with MB or PQ. Only males treated with MB during the prenatal period and with PQ during adulthood showed a significant reduction of locomotor activity (95%) and a selective DA loss in the pars compact of the substantia nigra. Exposure to specific pesticides during the prenatal period can produce a limited but selective amount of lesions in the nigrostriatal system. These developmental lesions might determine an enhanced susceptibility that, in combination with later exposure to other toxicants (PQ), may be involved in the induction of PD during aging.

The results of experimental studies on the role of pesticides are concordant with the results of epidemiologic studies. A series of epidemiologic studies have pointed out the importance of pesticides, in particular insecticides and fungicides. The problem with these studies is that they were mainly case–control, with a limited number of cases and without biologic measurement of the exposure. Most of the studies were based on retrospective collection of data with questionnaires (Checkoway and Nelson 1999). The only prospective study is the cohort study of Japanese men in Hawaii, originally established for the investigation of risk factors: 7,986 Japanese–American men born between 1900 and 1919 were enrolled in the longitudinal Honolulu Heart Program, with a median length of follow-up of 27 years. The relative risk of PD was 1.9 (95% CI, 1.0–3.5) for men who had worked more than 20 years on a plantation compared with men who never worked in a plantation (sugarcane and/or pineapple). The age-adjusted incidence of PD was higher in men exposed to pesticides than in men who never did plantation work (Petrovitch et al. 2002).

A recent meta-analysis on environmental risk factor and PD was performed using a large number of studies (16 studies for living in rural area, 18 studies for well water drinking, 11 studies for farming and 14 studies for pesticides) (Priyadarshi et al. 2001). Restricting the analysis to studies in the United States, the combined OR was 1.44 (95% CI, 0.92–2.24) for well water; 2.17 (95% CI, 1.54–3.06) for rural residence; 1.72 (95% CI, 1.2–2.46) for the combined exposure of living on a farm, farming, and exposure to animals; and 2.26 (95% CI, 1.95–2.39) for exposure to pesticides. The highest change in risk was for subjects living in rural areas for more than 40 years (OR = 4.9; 95% CI, 1.4–18.2). This might indicate that a cumulative exposure over time is needed but may also suggest that exposure during early life is important. In a study conducted in Saskatchewan, Canada, 20 of 22 cases of early-onset PD had exclusively rural exposure and drank well water during the first 15 years of life (Rajput et al. 1987). Well water consumed in childhood could be considered a potential vehicle for a toxic exposure. Analysis of pesticides and of metals in the well water did not reveal the role of any specific toxicant in this Canadian study.

Neurotoxicants such as MB, rotenone, and PQ interact with mitochondrial complex I (Dawson and Dawson 2003). Reduced activity of complex I has been described consistently in PD subjects (Schapira et al. 1998). A possible interaction between environmental risk factor and genetic polymorphism in mitochondrial genes and in genes involved in detoxification of metabolites has been intensively searched, with inconsistent results (Tan et al. 2000). The aggregation of α-synuclein and the subsequent inhibition of proteasome could be the consequence of complex I reduction in activity and may determine the death of DA neurons. The protection of proteasome function is one of the hypothesized roles of parkin (Feany and La Spada 2003).

Heterozygote mutations in the Parkin gene have been associated with late-onset PD (Oliveira et al. 2003). These parkin heterozygote mutations or other mutations could act as susceptibility genes in PD in conjunction with environmental risk factors, including neurotoxicants. Combined treatment with the two neurotoxicants PQ and Mn^2+^-ethylenebis-dithiocarbamate shows enhanced toxicity only in mice expressing human α-synuclein (Thiruchelvam et al. 2004). Only a mutant strain (hm^2^–α-Syn-39) presented enhanced vulnerability to neurotoxicants. This specific gene–environment interaction could be the source of different responses to neurotoxicants in different mice strains and in humans as well.

In conclusion, epidemiologic data suggest that the use of pesticides or surrogates of pesticide exposure increase the risk of PD. However, no epidemiologic studies specifically address the issue of toxic exposure early in life.

## Coffee, Smoking, and Personality Trait

In a recent meta-analysis, Hernan et al. (2002) analyzed 44 case–control studies and 4 cohort studies on smoking and 8 case–control studies and 5 cohort studies on coffee drinking. The risk of PD was 60% lower among smokers than among nonsmokers and about 30% lower in coffee drinkers than in non-coffee drinkers. Smoking and coffee are complex exposures, but the primary candidate substances for the protective effects are nicotine and caffeine, respectively. There is some evidence to support a protective effect of caffeine on the DA system. Mice treated with caffeine before exposure to MPTP had reduced DA cell loss compared with mice unexposed to caffeine (Chen et al. 2001). The action of caffeine seems to be mediated through a blocking action on adenosine-2 receptors (Schwarzschild et al. 2002). Similarly, in animal models, nicotine increases the presence of factors that have a neuroprotective effect on DA neurons (Maggio et al. 1998). Nicotine is an antioxidant (Ferger et al. 1998) and is an inhibitor of monoamine oxidase B that activates MPTP neuorotoxicity (Yong and Perry 1986). Nevertheless, a different biologic basis for these findings cannot be excluded.

A premorbid personality in PD patients has been described in several studies (Paulson and Dadmehr 1991; Todes and Lees 1985). Smoking and coffee drinking may be surrogates of an underlying personality trait or behavior and be related to a characteristic pre-parkinsonian personality (Marder and Logroscino 2002). Smoking and coffee consumption generally begin in early adulthood. Subjects who later develop PD may be less likely to have experiences that are associated with novelty or that are socially inappropriate (Bharucha et al. 1986; Ward et al. 1984). In a case–control study conducted with data from the Rochester Epidemiology Project, inverse associations were reported between PD and tobacco chewing, snuff use, and alcoholism (Benedetti et al. 2000). These exposures may represent patterns of unusual behavior and may depend on the presence of a specific personality trait.

Personality traits are established quite early in life, and it is known that specific personality traits are genetically related to the DA system (Ebstein et al. 1996). Twin studies are useful for trying to disentangle environmental and genetic risk factors. In twins discordant for PD, the twins who did not develop PD smoked more than their twin (Tanner et al. 2002). Interestingly, this effect was more pronounced in monozygotic twins. The effect remained even when the analysis was conducted 10 years before the disease onset. This study suggests a true biologic effect of smoking, but a further effect of other traits that are present even 20 years before the disease onset cannot be excluded. In a nested case–control study from Kaiser Permanente on 626 subjects and 1,245 controls, Van Den Eeden et al. (2002) classified subjects according to their personality traits. The protective effect of smoking was present even when restricting the analysis to individuals who scored high for introversion and depression. Among these subjects, smoking reduced the risk of PD by 50%.

In the EUROPARKINSON Collaborative Study, the protective effect of smoking was present only in early-onset PD (onset before age 50) but not in late-onset PD (Tzourio et al. 1997) Similar findings had been previously reported in the Northern Manhattan Aging Study in New York (Mayeux et al. 1994).

None of the previous studies looked at the age when subjects began to smoke. In data from the Nurses Health Study and the Health Professional Study, subjects who started to smoke early (before age 20) had a stronger reduction in risk, independent of future smoking behavior (A. Ascherio, personal communication). This observation suggests that if smoking is not causal, the other possible cause should already be present in the causal pathway before age 20.

Again, many of these studies suggest that the analysis of behavior in early adulthood could be key to disentangling the role of smoking, drinking coffee, and personality traits on the occurrence of PD.

## Familial Aggregation Studies

In early-onset PD, the mode of inheritance is autosomal dominant or recessive (Golbe et al. 1990; Kitada et al. 1998). The role of genes and the type of transmission are less clear for late-onset PD. It is well known that subjects with a family history of PD have an increased risk of disease (Lazzarini et al. 1994; Marder et al. 1996; Payami et al. 1994). Only a few of these studies have been population based, and none had complete genealogic information. The population-based design is important to avoid ascertainment bias because relatives of subjects with the disease are more likely to seek medical attention, and therefore families with more than one family member are more likely to be included. A recent study from Iceland coupled the genealogic information with a population-based study design (Sveinbjornsdottir et al. 2000). In Iceland a computerized database contains genealogic information for 11 centuries, including information on all the living Icelanders. PD cases (772 patients) were identified from several sources; 560 had late-onset PD. The risk ratio for PD was 6.7 for siblings, 3.2 for offspring, and 2.7 for nephews and nieces of patients with late-onset PD. The authors concluded that the pattern of inheritance was consistent with both genetic and environmental factors. In a subsequent comment on this study, it was noticed that the risk ratio for siblings and offspring was different, whereas the offspring and nieces and nephews had similar risk (de la Fuente-Fernandez and Calne 2001). This pattern of inheritance was not consistent with simple vertical or genetic clustering. These data might be explained by the presence of two birth cohorts (one for siblings and one for offspring and nephews and nieces) that had similar risk because of similar exposure in the same period of their life to some environmental risk factor. The results of the Icelandic study support the idea that environmental risk factors acting at different points in time during the life span (including early life) are critical for the development of PD.

There is evidence that some reported familial cases may be due to environmental factors. The age pattern within families with multiple cases can help to determine if there is a substantial involvement of environmental risk factors over a specific period of time (Calne et al. 1987). If an environmental exposure is present, a similar time of onset should be present in cases of the same family. If a genetic effect is the main causal factor, then a similar age of onset in different generations or a pattern of age of onset is expected. In six Canadian families with multiple cases, the time interval between onset of symptoms within the same family was relatively short (1–8 years), whereas the mean difference in age of onset of PD in children and parents in this series was 25.2 years. Also, no correlation was found between degree of consanguinity and occurrence of disease. This report underscores the importance of environmental risk factors in some of the familial PD cases.

In a case–control study conducted in Spain with 299 PD patients and 295 controls, the age of onset of PD was found to correlate with maternal age at birth of the patient, but not with paternal age. Both mothers and fathers had increased risk of PD compared with parents of controls (de la Fuente-Fernandez 2000). The author hypothesizes that environmental exposure may cause mitochondrial change in the mother that can then be transmitted to the child through the ovum. In this study, it is particularly interesting that the negative correlation of PD age of onset with maternal age was present also in PD probands with an affected father. This study suggests that environmental factors operating before birth may have a role in PD, even if this does not exclude the role of a genetic predisposition.

## Conclusions

There is experimental evidence that PD and other neurologic diseases related to aging may have their origin during pregnancy or in early life, including young adulthood. The epidemiologic studies exploring this hypothesis are few, and the most were conducted many years ago. Similar to that for cancer and cardiovascular disease, the etiologic model for PD has explored mainly risk factors associated with adult life or in many cases after the age of 65 (Kuh and Ben-Shlomo 1997). Cohort studies exploring neurologic disease related to aging, with few exceptions, are based on cohorts of subjects 65 or more years of age. The limitations of the case–control approach when exploring questions on exposures that happened decades before disease onset are well known.

The role of aging in the pathogenesis of PD is not clear, but recent evidence suggests that it is less important than was thought in the past. In a recent neuropathologic study there was no significant loss of tyrosine hydroxylase positive neurons in the substantia nigra in normal subjects (44– 80 years of age). This result suggests that normal aging is not accompanied by significant catecholaminergic cell loss (Kubis et al. 2000). If these data are confirmed, it is conceivable that risk factors that have a long latency or a slow effect could be important for late-onset PD, independent from a specific effect of aging. PD could be “programmed” *in utero* according to the Barker hypothesis for chronic diseases (Kuh and Ben-Shlomo 1997), and aging may be only a period where there is a second hit or an interaction with other processes. The risk factors in early life could determine an increased risk for PD, an earlier age of onset, or a different clinical course. Testing these pathogenetic models should be one of the future focuses of neuroepidemiologic research. To investigate the role of neurodevelopment on neurodegeneration, investigators must develop new methods to characterize the early-life environment of the elderly. The alternative strategy is to build up new cohorts or use existing cohorts that examine the whole life span, as in the Barker approach to study cardiovascular diseases.
